# One year mortality after pediatric hydrocephalus treatment: a comparative analysis of endoscopic third ventriculostomy and ventriculoperitoneal shunt

**DOI:** 10.3389/fsurg.2025.1538899

**Published:** 2025-05-22

**Authors:** Ahmad Alali, Wesam Alkabouni, Viktoria Aretz, Timo Volpert, Yollam Makanjira, Martin Kampeni, Thomas Kapapa, Patrick Kamalo

**Affiliations:** ^1^ Department of Neurosurgery, Ulm University Hospital, Ulm, Germany; ^2^ Blantyre Institute of Neurosurgical Sciences, Blantyre, Malawi

**Keywords:** hydrocefalus, ventriculo peritoneal shunt, ETV = endoscopic third ventriculostomy, Malawi, neurosurgery

## Abstract

**Background:**

Management options for hydrocephalus have increased to include endoscopic third ventriculostomy with or without choroid plexus cauterization (ETV ± CPC) in addition to traditional ventrikuloperitoneal shunting (VPS). This study evaluates the mortality and complications of these procedures in pediatric hydrocephalus, offering insights for clinical decision-making in a low-income country context.

**Methods:**

We retrospectively reviewed the operating theatre registry for infants under 1 year of age who underwent initial hydrocephalus surgery in a tertial sub-Saharan hospital in 2021. Follow-up was conducted for up to 1 year after surgery, confirming the patient's vital status (alive or dead) through hospital visits, contact information, and medical records. Descriptive analyses evaluated outcomes (mortality and complications), and survival was assessed using the Kaplan–Meier method with log-rank testing.

**Results:**

A total of 127 patients were included, with 71 males (55.91%). Complete 1-year follow-up data was available for 94 (74%) patients. Of these, 35 (37.23%) underwent ETV ± CPC and 59 (62.77%) underwent VPS. The one-year survival rate was 80% (95% CI: 66.75%–93.25%) for those treated with ETV ± CPC as a definitive treatment and 78% (95% CI: 67.43%–88.57%) for those who received VPS. There was no statistically significant difference in survival rates between the two groups (Log-Rank test *p* = 0.809). Shunt sepsis occurred in 6 patients (10.16%, 95% CI: 2.45%–17.87%). The majority of surgical complications occurred within the first 3 months following surgery, including shunt dysfunction in 4 VPS patients (6.7%, 95% CI: 0.32%–13.08%) and failed ETV in 10 patients (22.2%, 95% CI: 8.43%–35.97%) of those who underwent primary ETV.

**Conclusion:**

ETV ± CPC and VPS demonstrated similar survival rates, with no significant statistical difference between the two methods. However, ETV ± CPC failure often required conversion to VPS, highlighting the importance of managing shunt-related complications like sepsis and dysfunction. Careful post-operative monitoring is essential for both procedures.

## Introduction

Hydrocephalus is defined as an abnormal increase in the volume of cerebrospinal fluid (CSF) in the ventricular system of the brain, often due to CSF flow disorders, including dysfunctional absorption or, less frequently, increased production (1). Infants typically present with progressive macrocephaly, while older children show signs of intracranial hypertension (2). Hydrocephalus can lead to various cognitive dysfunctions, including deficits in attention, executive function, memory, visual-spatial abilities, and language, along with behavioral problems (1). If left untreated or inadequately managed, hydrocephalus can result in significant morbidity and mortality, particularly in infants.

The classic understanding of hydrocephalus as primarily caused by CSF flow obstruction is evolving to include models that incorporate cerebral pulsations, brain compliance, and newly characterized water transport mechanisms (2). This complexity underscores the importance of selecting appropriate treatment strategies. The two main surgical options for managing hydrocephalus are ventriculoperitoneal shunt insertion (VPS) and endoscopic third ventriculostomy (ETV), the latter often paired with choroid plexus cauterization (CPC) (ETV ± CPC) (3, 4). If the primary treatment fails, other types of shunts, such as ventriculo-atrial or ventriculo-pleural, may be considered (5, 6).

Ventriculoperitoneal shunt (VPS) remains the most widely used treatment, particularly in children (7). However, it is associated with significant complications, including shunt blockages, infections, and abdominal wound complications, which often necessitate repeated surgeries and lead to increased morbidity (7). The majority of these complications occur within the first 30 days post-operatively (8), contributing to high short-term mortality and the need for ongoing surgical intervention. As such, identifying alternatives to VPS that reduce complication rates and mortality is critical in resource-limited settings.

In recent years, ETV combined with CPC has shown promising results, particularly in Africa, where success rates have exceeded those seen in developed nations (9). When ETV/CPC is successful, patients often achieve shunt independence, which is associated with fewer complications and potentially lower mortality rates. Studies suggest that ETV/CPC is more successful in infants older than one month, particularly when hydrocephalus is secondary to myelomeningocele or aqueductal obstruction (10). These findings suggest that ETV/CPC may offer significant advantages over VPS, particularly in settings where access to follow-up care and repeated surgeries is limited.

Given the high prevalence of hydrocephalus in low-resource settings like Malawi, and the substantial risks associated with shunt complications, understanding which treatment option leads to better survival outcomes are critical. This study aims to compare the mortality rates of children under the age of one treated with ETV ± CPC as the first-line treatment vs. VPS at Queen Elizabeth Central Hospital (QECH) in Blantyre, Malawi. The results could have significant implications for clinical decision-making, potentially guiding the choice of surgical interventions in similar settings and ultimately improving patient outcomes by reducing mortality and treatment complications.

## Methods and material

This was a retrospective study using medical records of children under the age of 1 year, treated for hydrocephalus in a tertiary hospital, specifically the Department of Neurosurgery in southern Malawi. We reviewed data collected from patients treated between January 2021 and December 2021. Patients were either treated with ETV ± CPC or VPS. Patients were included if they were under one year of age and diagnosed with hydrocephalus. Diagnosis was made based on a combination of clinical presentation, such as progressive macrocephaly or signs of intracranial hypertension, and confirmed through neuroimaging (ultrasound, CT, or MRI). The clinical assessments were performed by either pediatricians or neurosurgeons, and the final diagnosis and treatment decisions were made by neurosurgeons. ETV ± CPC was the first-line treatment for patients with hydrocephalus, while VPS was used as the default alternative. The choice between these two treatment options was determined by several factors, including the patient's age, etiology of hydrocephalus, and anatomical suitability for ETV. ETV ± CPC was preferred for infants over one month old and those with non-communicating hydrocephalus, where the ventricles allowed safe endoscopic access. VPS was selected in cases where ETV ± CPC was deemed unsuitable, such as in patients with communicating hydrocephalus or those with anatomical constraints that would make endoscopic treatment less likely to succeed. We excluded patients if they fulfilled any of the following criteria:

(a) Primary treatment with other modalities (e.g., CPC only, external ventricular drain).
(b) Inadequate one-year data follow up (e.g., no contact possible, no adequate survival data).

Whether any of the included patients had received external ventricular drainage (EVD) prior to definitive treatment with ETV ± CPC or VPS could not be determined, as this information was not documented in the available medical records. The data were collected using the local patient data system, the Blantyre Institute of Neurological Sciences Electronic Medical Records System (BINS EMRS). Data on patients' survival status were collected by checking the BINS EMRS for any record of patient contact 365 days after the operation. “Survival” was defined as having confirmed documentation of the patient being alive one-year post-operation. For patients who died within the one-year follow-up period, we documented the date of death. However, causes of death were not documented in most cases. For patients with incomplete survival information one year after surgery, efforts were made to contact them or their families up to three times. If no contact was successful, the time in days from the surgery to the last known contact in the BINS EMRS was recorded as censored data, and these cases were included in the Kaplan–Meier survival analysis. We excluded patients without adequate vital status from the one-year-survival-analysis statistics. BINS EMRS was also used to identify subsequently operations following the definitive surgery, like VP shunt removal/revision, Endoscopic Cyst Fenestration and Ventriculoscopy.

The ETVs and CPCs are performed using a re-usable flexible neuro-endoscope (Karl-Storz, Germany), disinfected in Cidex OPM (ASP, USA). Successful fenestration of the floor of the third ventricle is defined as a fenestration posterior to the posterior clinoid, and anterior to the mamillary bodies, with exposure of the bare basilar artery, and its branches, and posterior to the membrane of Lilliquist (11). Using the flexible scope, the neurosurgeon proceeds down into the prepontine space to visualize the lower cranial nerves and the vertebral arteries uniting to form the basilar artery. After controlling for correct fenestration, the neurosurgeon carefully removes the endoscope and closes the scalp.

VPS as the default alternative for treatment of hydrocephalus is a procedure to connect the ventricular system of the brain to the peritoneal cavity. For children in QECH most often the lateral ventricle of the non-dominant right brain side is punctured. A flexible silastic (a combination of silicon and plastic) tube placed in subcutaneous tissue connects the two cavities. Successful VPS can be checked by making sure CSF drops from the peritoneal edge of the silicon tube. For VPSs', a hydrocephalus shunt system from Chhabra, India was used for all patients.

Statistical analyses included descriptive statistics for patient characteristics and a comparison of survival rates between the ETV ± CPC and VPS groups. Quantitative parameters were presented by mean, standard deviation, median, and range. Qualitative parameters were represented by absolute and relative frequencies. *T*-test, ANOVA and non-parametric tests were performed considering the size of the subsets for explorative tests. Survival analysis was based on the time from the definitive surgical procedure to either the patient's death or the 1-year follow-up mark. Patients with incomplete or inadequate follow-up data were excluded from the analysis. For patients who died within the follow-up period, the survival time was measured from the date of surgery to the date of death. All analyses were performed using IBM SPSS Statistics, version 29.0 (IBM Corp., Armonk, NY, USA). The level of significance was set at *p* ≤ 0.05.

## Study results

We found a total of 127 patients, operated for treatment of hydrocephalus in the data register. 123 patients underwent surgery either for VPS or ETV ± CPC. Four patients had neither VPS nor ETV as definitive surgery during the study period (one External Ventricular Drainage, one Removal of Ventriculo-Peritoneal Shunt, one Subgaleal Shunt, one Wound Debridement). The case involving wound debridement was included due to documented hydrocephalus as the indication for surgery, though the exact reason (whether infection-related or not) remains uncertain. The cause of hydrocephalus was not documented in most cases. Among the documented cases, congenital hydrocephalus and post-infection hydrocephalus were the most observed causes. Specifically, 13 cases were documented as congenital hydrocephalus, 4 cases were due to a cyst in the ventricular system, 2 cases were post-infection hydrocephalus, and 1 case was combined with an occipital encephalocele. Based on available radiological and intraoperative findings, 17 of these 20 cases were classified as obstructive hydrocephalus (congenital and cyst-related), and 3 cases as communicating hydrocephalus (2 post-infectious, 1 encephalocele-associated). In the remaining 107 patients (84.25%), the cause of hydrocephalus was not documented.

**Figure 1:**
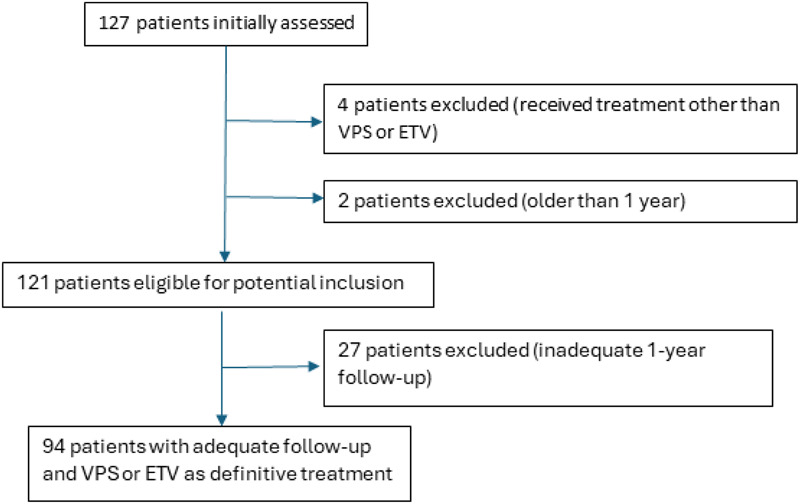
Treatment pathway for all 127 patients presenting to QECH.

From 127 patients initially assessed, 4 patients were excluded due to receiving treatment other than VPS or ETV, and 2 patients were excluded for being older than 1 year. This left 121 patients eligible for potential inclusion. Of these, 27 patients were excluded due to inadequate 1-year follow-up, resulting in 94 patients with adequate follow-up who received VPS or ETV as definitive treatment. The treatment pathway of all 127 patients is illustrated in Figure 1.

From the 94 patients with adequate 1-year follow-up, 59 (62.77%) were treated with VPS, and 35 (37.23%) underwent ETV ± CPC. A survival analysis using the Log Rank test (Mantel-Cox) showed no significant difference between the two treatment groups (*p* = 0.809). The mean age of the patients was 153.34 days (interquartile range 89.00–205.50) in the overall cohort. Patients who received VPS had a mean age of 144.80 days and were not significantly older than ETV patients, who had a mean age of 167.74 days (Mann–Whitney *U* test: *p* = 0.224). Overall, 36.8% (42 patients) of all patients were female. Among those who received VPS, 47.5% (28 patients) were female, whereas 40% (21 patients) of the patients treated with ETV ± CPC were female (Pearson's chi-square test: *p* = 0.495).

Among the 94 patients, 65 (69.15%) were under the age of six months at the time of surgery. Of these younger patients, 45 (69.23%) were treated with VPS, and 20 (30.77%) were treated with ETV ± CPC. Regarding mortality, 11 out of 45 patients (24.44%) treated with VPS and 3 out of 20 patients (15%) treated with ETV ± CPC died during the follow-up period.

In contrast, 29 (30.85%) patients were older than six months. In this group, 14 (48.28%) were treated with VPS, and 15 (51.72%) underwent ETV ± CPC. Mortality in the older group was lower, with 3 out of 14 patients (21.43%) treated with VPS and 2 out of 15 patients (13.33%) treated with ETV ± CPC dying during the follow-up period.

Table 1 presents data for the 94 patients (100%) with adequate one-year follow-up. Among these, 35 patients (37.23%) underwent treatment with ETV ± CPC, while 59 patients (62.77%) were treated with VPS. The overall one-year mortality rate among all eligible patients was 21.28% (95% CI: 13.00%–29.55%). In the ETV ± CPC group, 7 patients (20%) died within the year. In contrast, the VPS group had a one-year mortality rate of 22.03%, with 13 out of 59 patients passing away within the year.

**Table 1:** Patients characteristics for 94 patients treated for hydrocephalus.

Characteristic	*N*	(%)
Number of patients	94	(100.00)
Males	52	(55.32)
Females	42	(44.68)
Median age in days (SD)	128.50	(87.712)
VPS	59	(62.77)
<6 months	45	(76.27)
>6 months	14	(23.73)
ETV ± CPC	35	(37.23)
<6 months	20	(57.14)
>6 months	*15*	(42.86)
Mortality	20	(21.28)
VPS mortality	13	(22.03)
ETV mortality	7	(20.00)
VPS	59	(62.77)
Shunt infection	6	(10.16)
Patients needed ≥1 operations	9	(15.25)
Primary ETV ± CPC	45	(47.87)
Failed ETV	35	(77.87)
ETV as definitive treatment	10	(22.22)

*N*, absolute frequency; %, relative frequency; SD, standard deviation; <, less than; >, greater than; VPS, ventriculoperitoneal shunt; ETV ± CPC, endoscopic third ventriculostomy with or without choroid plexus cauterization.

From the initial 45 patients who underwent ETV ± CPC, 10 required conversion to VPS. Table 2 shows an overview of the number of patients who died and survived in both treatment methods. Among the remaining 35 patients who continued with ETV ± CPC, 3 patients (8.57%) needed one or more follow-up operations. For patients treated with VPS, 9 out of 59 (15.25%) required one or more follow-up operations during the observed one-year survival period.

**Table 2:** Summary of the case processing.

Operation	Total numbaer	Died	Censored	
			Survived	Percentage
VPS	59	13	46	78.0%
ETV ± CPC	35	7	28	80.0%
Total	94	20	74	78.7%

**Figure 2:**
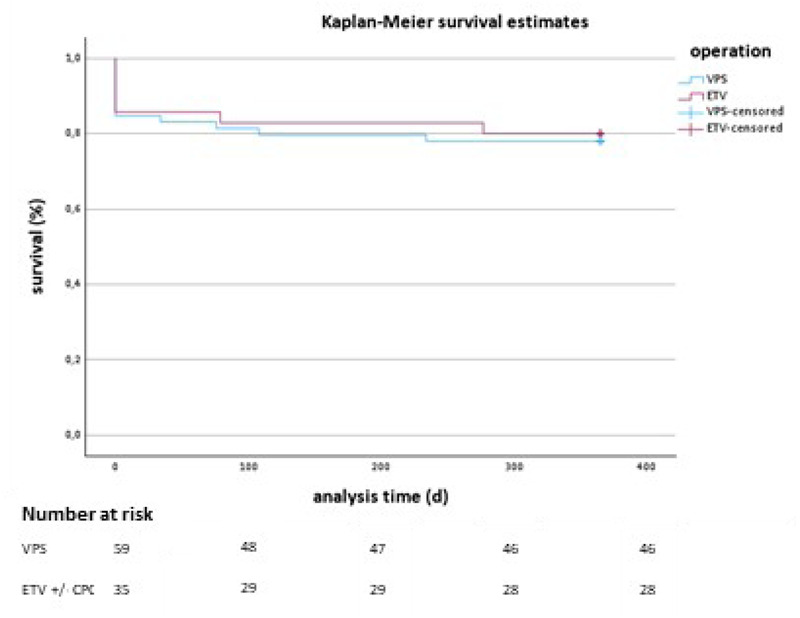
Kaplan–Meier survival curves for patients treated with VPS vs. ETV ± CPC.

The Kaplan–Meier survival analysis for VPS and ETV treatments shows similar survival trajectories, with no statistically significant difference between the groups (*p* = 0.809 from the Log Rank test). Figure 2 displays the Kaplan–Meier survival curves comparing both treatment groups. The *x*-axis displays the analysis time in days, and the *y*-axis shows the survival rate as a percentage. Both groups start with an initial drop in survival rates shortly after treatment, but they stabilize at a similar survival percentage for the remainder of the observation period. The blue line represents the VPS treatment group, and the red line represents the ETV treatment group. Vertical tick marks on each line indicate censored data points, showing where patients were lost to follow-up or had other forms of dropout.

By the end of the one-year follow-up period, both groups show comparable survival rates, with no distinct separation between the two curves. This visual trend supports the statistical findings, suggesting that neither VPS nor ETV treatment demonstrates a significant survival advantage.

## Discussion

In our study, we found a total mortality rate of 21.28% (95% CI: 13.00%–29.55%). For patients treated with VPS, the mortality rate was 22.03%, and 20% for those treated with ETV ± CPC. 78.72% of patients with adequate follow-up were alive one year after definitive surgical treatment (78.00% for VPS, 80.00% for ETV ± CPC). The one-year event-free rate was 75.53% (95% CI: 66.84%–84.22%).

Our findings show higher overall mortality rates compared to the study by Chimaliro et al. (12), which assessed the mortality and one-year complication rate after treatment of hydrocephalus in Malawi, reporting a mortality rate of 15% for both VPS and ETV. Their one-year event-free rate was 67.2% for both treatments. It is important to note that the patient demographics, clinical settings, and treatment protocols may vary significantly between our study and that of Chimaliro et al., which could explain the differences in absolute mortality rates observed.

Chimaliro et al., as well as Limbrick et al. (13), concluded that VPS and ETV ± CPC have equivalent mortality rates in clinical etiological studies. Our findings demonstrate similar results. We found comparable mortality rates for both VPS and ETV ± CPC (22.03% vs. 20%). Additionally, our one-year event-free rate was higher compared to Chimaliro et al. (12) (75.53% vs. 67.2%). Also, fewer patients in our study population had one or more follow-up operations compared to Chimaliro et al. (23.40% vs. 27.84%). The similar mortality rates of VPS and ETV ± CPC observed in our study suggest comparable outcomes for both treatment approaches. The optimal treatment for hydrocephalus remains controversial. While ETV is often used for obstructive hydrocephalus in children older than 2 years and adults, VPS placement continues to be the standard of care (2).

Both ETV and VPS are associated with complications. Shunt failure, typically due to mechanical obstruction, necessitates intervention in 40% of children within the first 2 years after placement (14). The rate of shunt infection is about 5%–9% per procedure (15, 16), mostly occurring within 3 months of surgery (17). 6 (10.16%) of the 59 patients who received VPS with adequate 1-year follow-up had a shunt infection. Chimaliro et al. reported a shunt infection rate of 21.4% (12). Shunt overdrainage can present acutely with subdural hygroma or hematoma, or chronically with slit-ventricle syndrome (18). In unselected cohorts, the incidence of ETV failure at 2 years is about 35% (19), with true incidence dependent on individual prognostic factors, especially age and cause of hydrocephalus (20). Most ETV failures occur within the first 6 months of surgery (20–22). Infection after ETV is less common than with shunt procedures, occurring in fewer than 2% of cases (23). For patients whose ETV ± CPC treatment has failed, the next step is to use VPS. Chimaliro et al. reported that once ETV failed, the ETV revision success rate was only 6% so that they don't recommend a second ETV ± CPC after the failed first one. 22.22% of our patient treated with ETV ± CPC needed a VPS. Chimaliro et al. reported a similar rate (12). 25% of the ETVs they performed failed and ended up with VPS.

Hydrocephalus is defined as any increase in cerebrospinal fluid in the skull (24). Walter Dandy classified hydrocephalus into obstructive and communicating types, a system still commonly used today (25). Ventriculoperitoneal shunt (VPS) remains the most widely used treatment, particularly in children (7). Endoscopic third ventriculostomy (ETV) is an effective alternative treatment for hydrocephalus, particularly for patients with noncommunicating hydrocephalus (26). The age of the child also has relevance to the success rate of ETV ± CPC. Studies have shown that therapy with ETV ± CPC of children under 6 months of age is more prone to fail (27). Laeke T et al. mentioned that the ETV ± CPC worked in 50% of cases in children less than 6 months of age after one year (28).

Our study demonstrated important results about the success of the treatment with an endoscopic intervention and with a shunt. We found an ETV success rate of 62.22%. From the initial 45 patients who underwent ETV ± CPC, 10 required conversion to VPS. Among the remaining 35 patients who continued with ETV ± CPC, 3 patients (8.57%) needed one or more follow-up operations. Chimaliro et al. mentioned that 50% of their patients were successfully treated with ETV ± CPC without needing a VP shunt (12). This rate is slightly lower than the one we found. Another study that also looked at ETV success rate, from 2020, is the one by Lepard JR et al. (29). Their title was evaluation and external validation of a new public health strategy for treating paediatric hydrocephalus in low-resource settings. Lepard JR et al. evaluated a new evidence-based treatment algorithm to reduce shunt-dependence in Uganda and Nigeria. Lepard JR. et al. found an ETV success rate of 54% which is also lower than our rate.

Another important aspect that we have not covered is the causes of hydrocephalus, as they are not documented in our data. Hydrocephalus can be classified as acquired or developmental hydrocephalus (30). Acquired hydrocephalus is commonly caused by hemorrhage, neoplasm, and infection, with hemorrhage being the most prevalent (31). The data on developmental hydrocephalus is very thin and more research is needed (32). Warf B.C. dealt with Pediatric hydrocephalus in East Africa in 2010 and mentioned that most hydrocephalus cases in children are due to preventable causes such as neonatal infections and vertebral dysplasia (33). Neonatal infections have not been definitively understood and research is still needed (34). With the help of public health measures, neonatal infections can be reduced and thus some decrease in hydrocephalus is expected (35).

From 127 patients, we excluded 4 (3.14%) patients due to receiving treatment other than VPS or ETV. Two (1.57%) patients were excluded for being older than 1 year. 27 (21.25%) patients were excluded due to inadequate 1-year follow-up. In the end, we could evaluate the data of 94 (74.01%) patients with adequate follow-up who received VPS or ETV as definitive treatment.

The results of our study differ in some aspects from those of other studies but are consistent in others. It is crucial to consider the quality and context of these comparisons. Our study's high follow-up rate enhances the reliability of our data, but variations in patient demographics, clinical settings, and methodologies must be acknowledged when comparing to other studies. For instance, while our mortality rates are higher than those reported by Chimaliro et al. (12), the differences in geographic and socioeconomic factors may influence these outcomes. Similarly, our shunt infection rates align with established ranges but highlight the importance of continuous monitoring and improved postoperative care. By critically evaluating these differences and similarities, we can better understand the nuances and limitations of our findings and contribute to the broader body of knowledge on hydrocephalus treatment.

It should be noted that mortality is high in the analyzed population, which may be due to geographical and socio-economic conditions. Additionally, while our study suggests that factors such as patient age, etiology of hydrocephalus, and anatomical suitability for ETV and ETV ± CPC influence treatment selection, it is not possible to determine whether the treatment method itself or external factors such as geographical and socio-economic conditions impact mortality. Due to the retrospective nature of this study, a direct comparison between the treatment groups is not possible. These aspects represent further limitations of our study.

We had no influence on the completeness and quality of the data, as the data were analyzed retrospectively. Many electronic files were not complete, which led to the exclusion of many patients. In addition, many interesting characteristics could not be studied. A further limitation is that it was not possible to determine whether any patients had undergone temporary cerebrospinal fluid diversion (e.g., EVD) prior to their definitive treatment, as this information was not available in the retrospective records. It should be noted that mortality is a surrogate factor, as death one year after surgery in a low-income country can have various causes beyond the surgical intervention. However, focusing on the one-year period reflects the likelihood of complications arising during this time. In the future, prospective studies on hydrocephalus treatment with high quality and complete information are of great importance. However, further research is needed for optimal hydrocephalus therapy, such as the role of neonatal infections as a cause of hydrocephalus development on the one hand and the development of postoperative shunt infections on the other.

## Conclusion

Further research is needed for optimal hydrocephalus therapy, such as the role of neonatal infections as a cause of hydrocephalus development on the one hand and the development of postoperative shunt infections on the other. Although the present study has limitations in terms of the retrospective nature and record deficiencies, it does give some insights into hydrocephalus management outcomes and the persistent all for more research and a comprehensive evaluation of management options.

## Data Availability

The original contributions presented in the study are included in the article/Supplementary Material, further inquiries can be directed to the corresponding author.
